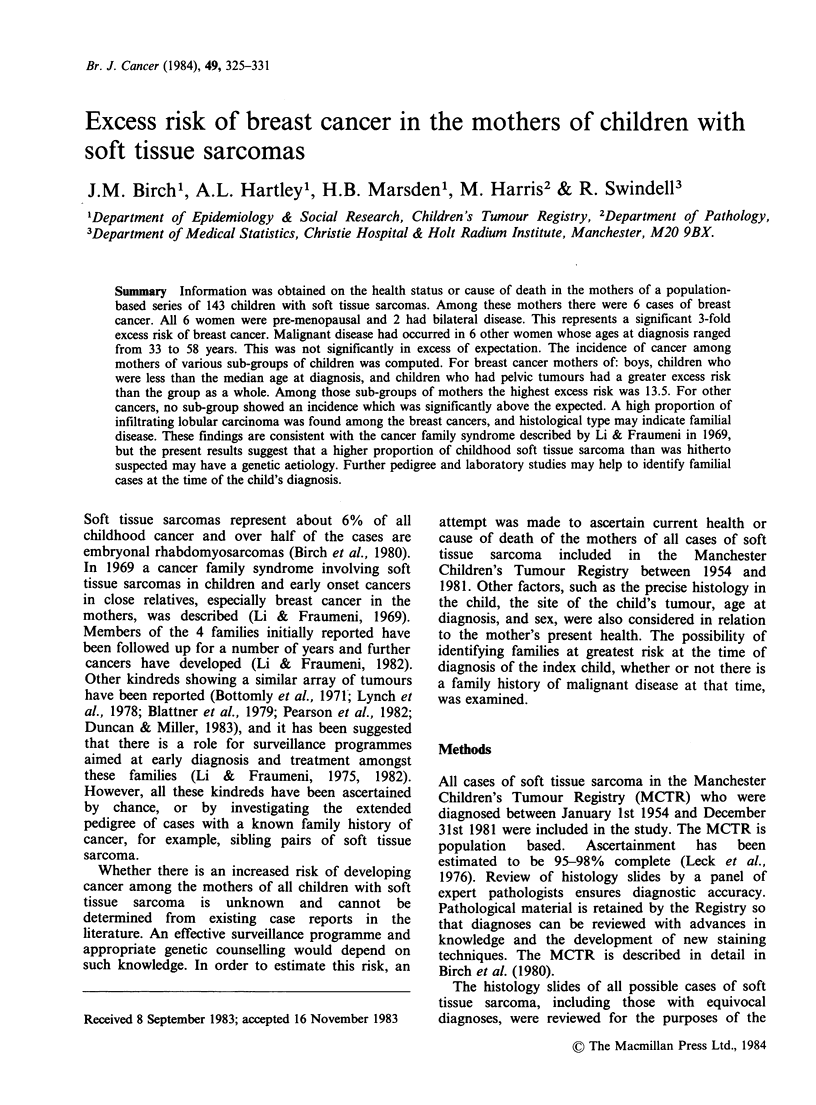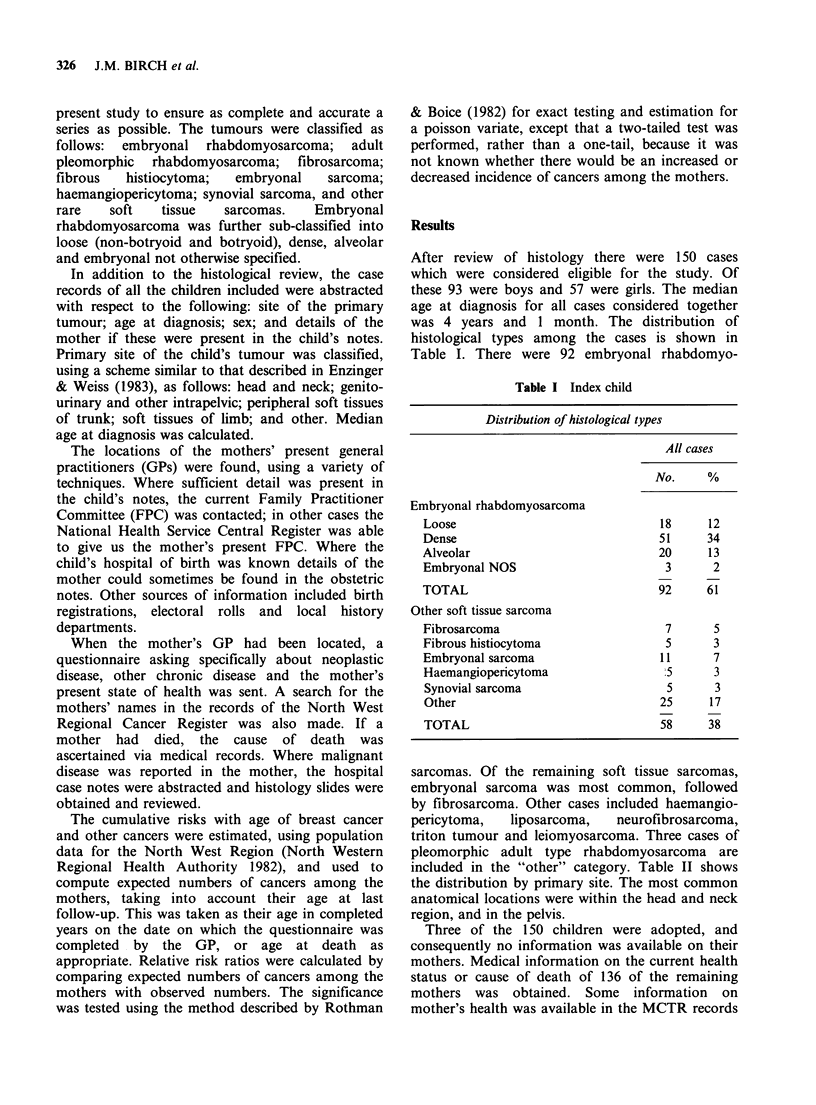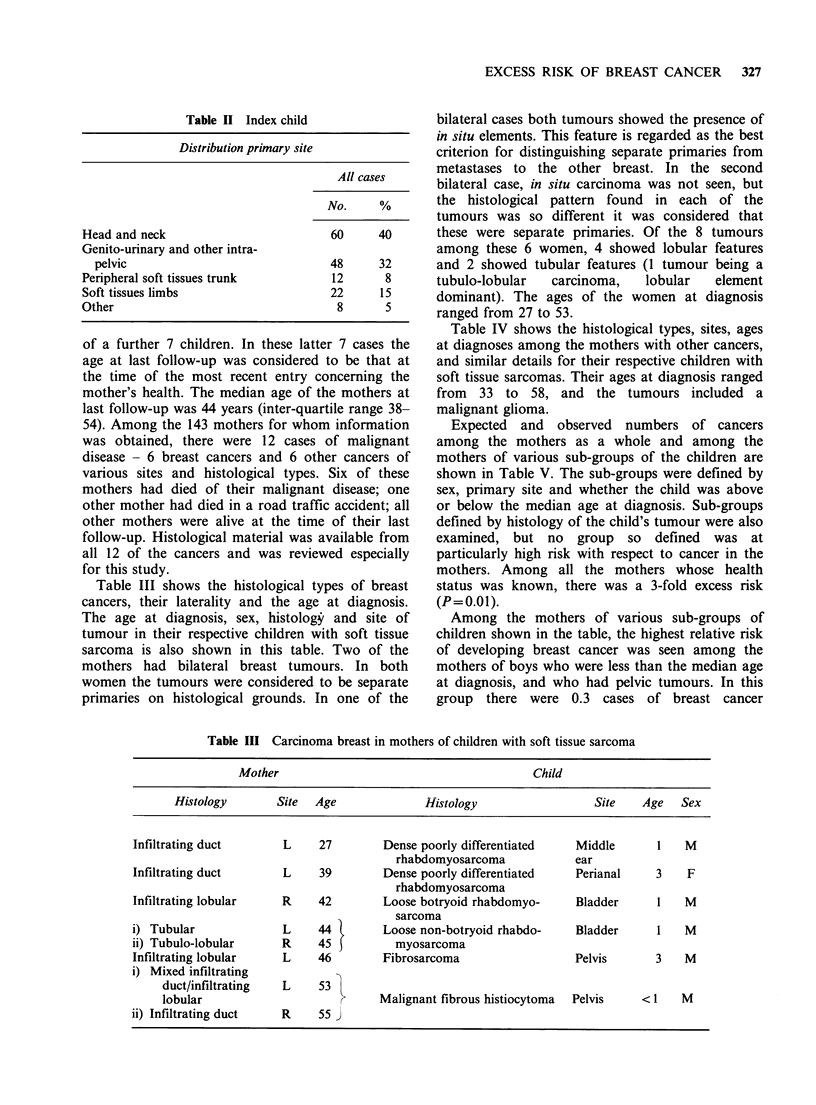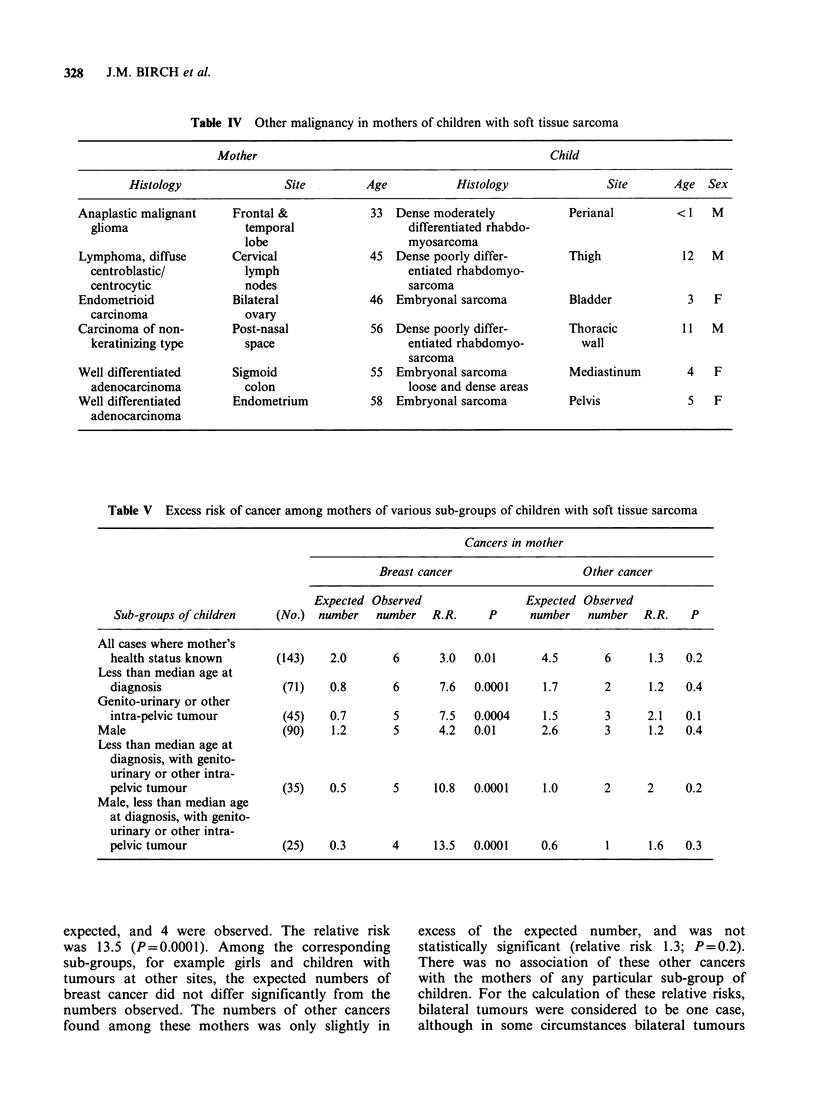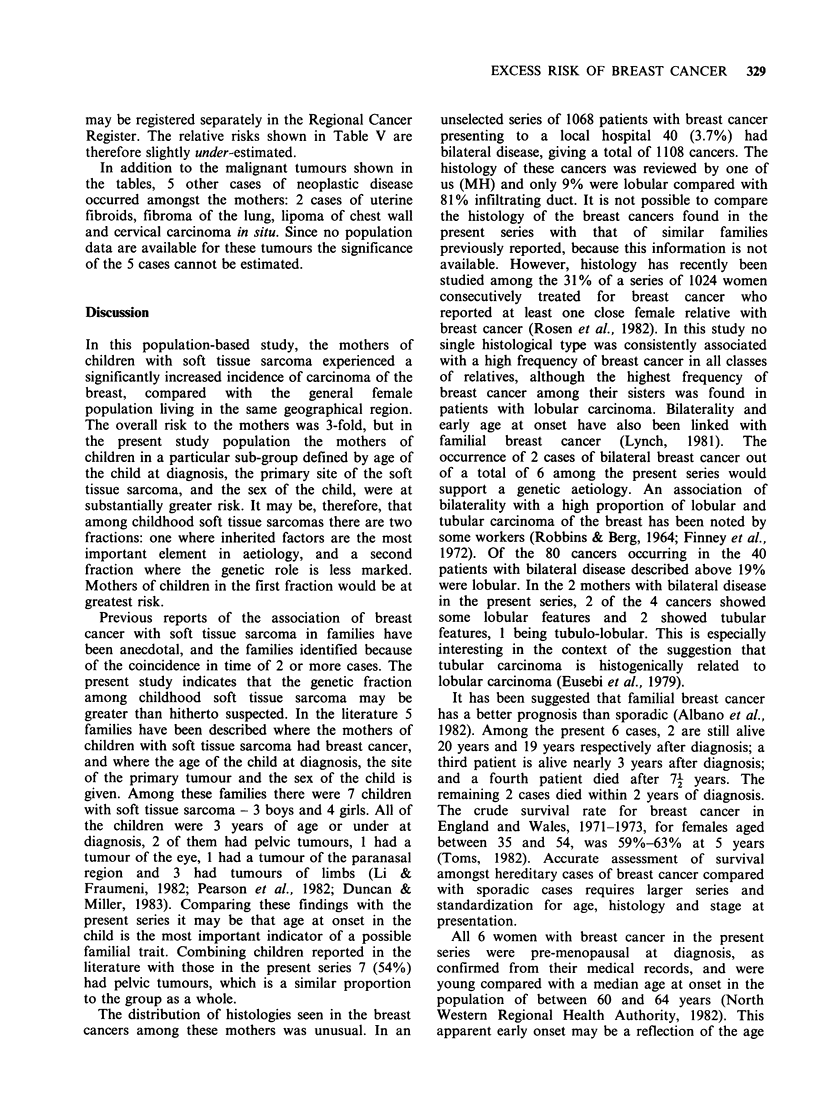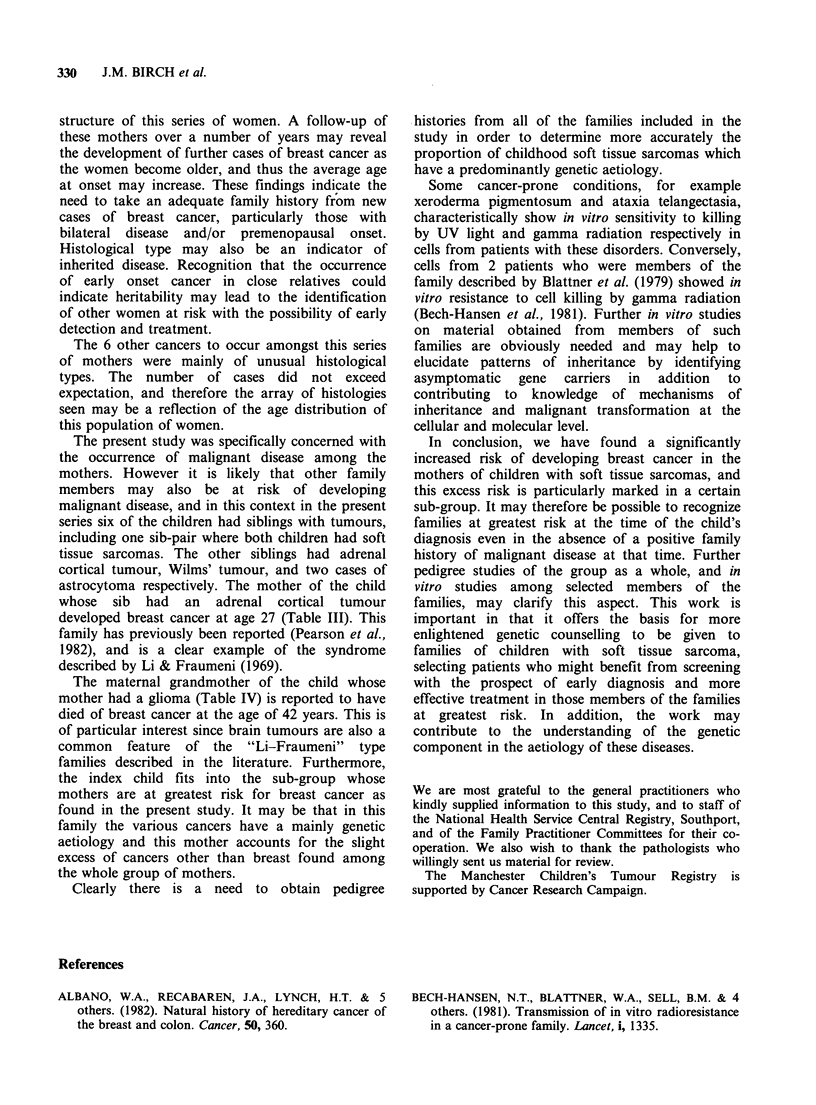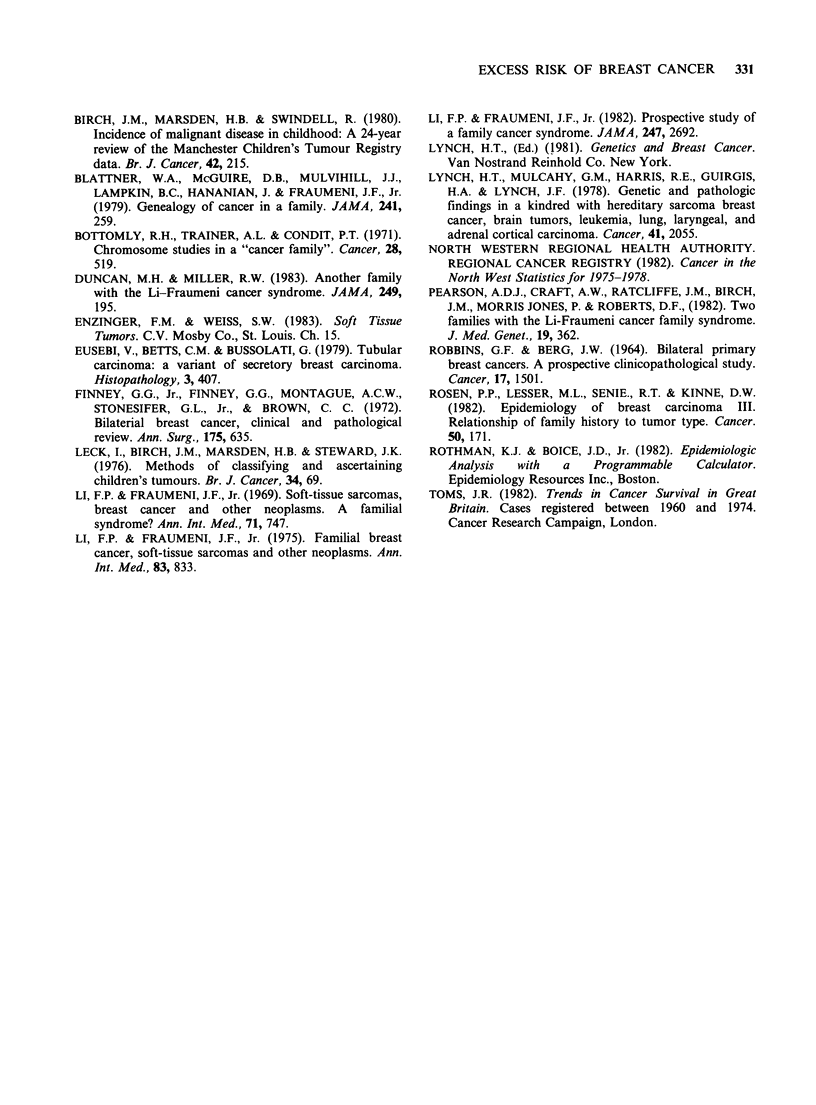# Excess risk of breast cancer in the mothers of children with soft tissue sarcomas.

**DOI:** 10.1038/bjc.1984.51

**Published:** 1984-03

**Authors:** J. M. Birch, A. L. Hartley, H. B. Marsden, M. Harris, R. Swindell

## Abstract

Information was obtained on the health status or cause of death in the mothers of a population-based series of 143 children with soft tissue sarcomas. Among these mothers there were 6 cases of breast cancer. All 6 women were pre-menopausal and 2 had bilateral disease. This represents a significant 3-fold excess risk of breast cancer. Malignant disease had occurred in 6 other women whose ages at diagnosis ranged from 33 to 58 years. This was not significantly in excess of expectation. The incidence of cancer among mothers of various sub-groups of children was computed. For breast cancer mothers of: boys, children who were less than the median age at diagnosis, and children who had pelvic tumours had a greater excess risk than the group as a whole. Among those sub-groups of mothers the highest excess risk was 13.5. For other cancers, no sub-group showed an incidence which was significantly above the expected. A high proportion of infiltrating lobular carcinoma was found among the breast cancers, and histological type may indicate familial disease. These findings are consistent with the cancer family syndrome described by Li & Fraumeni in 1969, but the present results suggest that a higher proportion of childhood soft tissue sarcoma than was hitherto suspected may have a genetic aetiology. Further pedigree and laboratory studies may help to identify familial cases at the time of the child's diagnosis.


Br. J. Cancer (1984), 49, 325–331

### Excess risk of breast cancer in the mothers of children with soft tissue sarcomas

J.M. Birch[1], A.L. Hartley[1], H.B. Marsden[1], M. Harris[2] & R. Swindell[3]

[1]*Department of Epidemiology & Social Research, Children's Tumour Registry,* [2]*Department of Pathology,* [3]*Department of Medical Statistics, Christie Hospital & Holt Radium Institute, Manchester, M20 9BX.*

**Summary** Information was obtained on the health status or cause of death in the mothers of a population-based series of 143 children with soft tissue sarcomas. Among these mothers there were 6 cases of breast cancer. All 6 women were pre-menopausal and 2 had bilateral disease. This represents a significant 3-fold excess risk of breast cancer. Malignant disease had occurred in 6 other women whose ages at diagnosis ranged from 33 to 58 years. This was not significantly in excess of expectation. The incidence of cancer among mothers of various sub-groups of children was computed. For breast cancer mothers of: boys, children who were less than the median age at diagnosis, and children who had pelvic tumours had a greater excess risk than the group as a whole. Among those sub-groups of mothers the highest excess risk was 13.5. For other cancers, no sub-group showed an incidence which was significantly above the expected. A high proportion of infiltrating lobular carcinoma was found among the breast cancers, and histological type may indicate familial disease. These findings are consistent with the cancer family syndrome described by Li & Fraumeni in 1969, but the present results suggest that a higher proportion of childhood soft tissue sarcoma than was hitherto suspected may have a genetic aetiology. Further pedigree and laboratory studies may help to identify familial cases at the time of the child's diagnosis.

Soft tissue sarcomas represent about 6% of all childhood cancer and over half of the cases are embryonal rhabdomyosarcomas (Birch *et al.*, 1980). In 1969 a cancer family syndrome involving soft tissue sarcomas in children and early onset cancers in close relatives, especially breast cancer in the mothers, was described (Li & Fraumeni, 1969). Members of the 4 families initially reported have been followed up for a number of years and further cancers have developed (Li & Fraumeni, 1982). Other kindreds showing a similar array of tumours have been reported (Bottomly *et al.*, 1971; Lynch *et al.*, 1978; Blattner *et al.*, 1979; Pearson *et al.*, 1982; Duncan & Miller, 1983), and it has been suggested that there is a role for surveillance programmes aimed at early diagnosis and treatment amongst these families (Li & Fraumeni, 1975, 1982). However, all these kindreds have been ascertained by chance, or by investigating the extended pedigree of cases with a known family history of cancer, for example, sibling pairs of soft tissue sarcoma.

Whether there is an increased risk of developing cancer among the mothers of all children with soft tissue sarcoma is unknown and cannot be determined from existing case reports in the literature. An effective surveillance programme and appropriate genetic counselling would depend on such knowledge. In order to estimate this risk, an attempt was made to ascertain current health or cause of death of the mothers of all cases of soft tissue sarcoma included in the Manchester Children's Tumour Registry between 1954 and 1981. Other factors, such as the precise histology in the child, the site of the child's tumour, age at diagnosis, and sex, were also considered in relation to the mother's present health. The possibility of identifying families at greatest risk at the time of diagnosis of the index child, whether or not there is a family history of malignant disease at that time, was examined.

Received 8 September 1983; accepted 16 November 1983

### Methods

All cases of soft tissue sarcoma in the Manchester Children's Tumour Registry (MCTR) who were diagnosed between January 1st 1954 and December 31st 1981 were included in the study. The MCTR is population based. Ascertainment has been estimated to be 95–98% complete (Leck *et al.*, 1976). Review of histology slides by a panel of expert pathologists ensures diagnostic accuracy. Pathological material is retained by the Registry so that diagnoses can be reviewed with advances in knowledge and the development of new staining techniques. The MCTR is described in detail in Birch *et al.* (1980).

The histology slides of all possible cases of soft tissue sarcoma, including those with equivocal diagnoses, were reviewed for the purposes of the

© The Macmillan Press Ltd., 1984

present study to ensure as complete and accurate a series as possible. The tumours were classified as follows: embryonal rhabdomyosarcoma; adult pleomorphic rhabdomyosarcoma; fibrosarcoma; fibrous histiocytoma; embryonal sarcoma; haemangiopericytoma; synovial sarcoma, and other rare soft tissue sarcomas. Embryonal rhabdomyosarcoma was further sub-classified into loose (non-botryoid and botryoid), dense, alveolar and embryonal not otherwise specified.

In addition to the histological review, the case records of all the children included were abstracted with respect to the following: site of the primary tumour; age at diagnosis; sex; and details of the mother if these were present in the child's notes. Primary site of the child's tumour was classified, using a scheme similar to that described in Enzinger & Weiss (1983), as follows: head and neck; genito-urinary and other intrapelvic; peripheral soft tissues of trunk; soft tissues of limb; and other. Median age at diagnosis was calculated.

The locations of the mothers' present general practitioners (GPs) were found, using a variety of techniques. Where sufficient detail was present in the child's notes, the current Family Practitioner Committee (FPC) was contacted; in other cases the National Health Service Central Register was able to give us the mother's present FPC. Where the child's hospital of birth was known details of the mother could sometimes be found in the obstetric notes. Other sources of information included birth registrations, electoral rolls and local history departments.

When the mother's GP had been located, a questionnaire asking specifically about neoplastic disease, other chronic disease and the mother's present state of health was sent. A search for the mothers' names in the records of the North West Regional Cancer Register was also made. If a mother had died, the cause of death was ascertained via medical records. Where malignant disease was reported in the mother, the hospital case notes were abstracted and histology slides were obtained and reviewed.

The cumulative risks with age of breast cancer and other cancers were estimated, using population data for the North West Region (North Western Regional Health Authority 1982), and used to compute expected numbers of cancers among the mothers, taking into account their age at last follow-up. This was taken as their age in completed years on the date on which the questionnaire was completed by the GP, or age at death as appropriate. Relative risk ratios were calculated by comparing expected numbers of cancers among the mothers with observed numbers. The significance was tested using the method described by Rothman & Boice (1982) for exact testing and estimation for a poisson variate, except that a two-tailed test was performed, rather than a one-tail, because it was not known whether there would be an increased or decreased incidence of cancers among the mothers.

### Results

After review of histology there were 150 cases which were considered eligible for the study. Of these 93 were boys and 57 were girls. The median age at diagnosis for all cases considered together was 4 years and 1 month. The distribution of histological types among the cases is shown in Table I. There were 92 embryonal rhabdomyosarcomas. Of the remaining soft tissue sarcomas, embryonal sarcoma was most common, followed by fibrosarcoma. Other cases included haemangiopericytoma, liposarcoma, neurofibrosarcoma, triton tumour and leiomyosarcoma. Three cases of pleomorphic adult type rhabdomyosarcoma are included in the "other" category. Table II shows the distribution by primary site. The most common anatomical locations were within the head and neck region, and in the pelvis.

**Table I** Index child

*Distribution of histological types*

| | *All cases* | |
|---|---|---|
| | *No.* | *%* |
| Embryonal rhabdomyosarcoma | | |
| Loose | 18 | 12 |
| Dense | 51 | 34 |
| Alveolar | 20 | 13 |
| Embryonal NOS | 3 | 2 |
| TOTAL | 92 | 61 |
| Other soft tissue sarcoma | | |
| Fibrosarcoma | 7 | 5 |
| Fibrous histiocytoma | 5 | 3 |
| Embryonal sarcoma | 11 | 7 |
| Haemangiopericytoma | 5 | 3 |
| Synovial sarcoma | 5 | 3 |
| Other | 25 | 17 |
| TOTAL | 58 | 38 |

Three of the 150 children were adopted, and consequently no information was available on their mothers. Medical information on the current health status or cause of death of 136 of the remaining mothers was obtained. Some information on mother's health was available in the MCTR records

**Table II** Index child

| *Distribution primary site* | *All cases* No. | *All cases* % |
|---|---|---|
| Head and neck | 60 | 40 |
| Genito-urinary and other intra-pelvic | 48 | 32 |
| Peripheral soft tissues trunk | 12 | 8 |
| Soft tissues limbs | 22 | 15 |
| Other | 8 | 5 |

of a further 7 children. In these latter 7 cases the age at last follow-up was considered to be that at the time of the most recent entry concerning the mother's health. The median age of the mothers at last follow-up was 44 years (inter-quartile range 38–54). Among the 143 mothers for whom information was obtained, there were 12 cases of malignant disease – 6 breast cancers and 6 other cancers of various sites and histological types. Six of these mothers had died of their malignant disease; one other mother had died in a road traffic accident; all other mothers were alive at the time of their last follow-up. Histological material was available from all 12 of the cancers and was reviewed especially for this study.

Table III shows the histological types of breast cancers, their laterality and the age at diagnosis. The age at diagnosis, sex, histology and site of tumour in their respective children with soft tissue sarcoma is also shown in this table. Two of the mothers had bilateral breast tumours. In both women the tumours were considered to be separate primaries on histological grounds. In one of the bilateral cases both tumours showed the presence of *in situ* elements. This feature is regarded as the best criterion for distinguishing separate primaries from metastases to the other breast. In the second bilateral case, *in situ* carcinoma was not seen, but the histological pattern found in each of the tumours was so different it was considered that these were separate primaries. Of the 8 tumours among these 6 women, 4 showed lobular features and 2 showed tubular features (1 tumour being a tubulo-lobular carcinoma, lobular element dominant). The ages of the women at diagnosis ranged from 27 to 53.

Table IV shows the histological types, sites, ages at diagnoses among the mothers with other cancers, and similar details for their respective children with soft tissue sarcomas. Their ages at diagnosis ranged from 33 to 58, and the tumours included a malignant glioma.

Expected and observed numbers of cancers among the mothers as a whole and among the mothers of various sub-groups of the children are shown in Table V. The sub-groups were defined by sex, primary site and whether the child was above or below the median age at diagnosis. Sub-groups defined by histology of the child's tumour were also examined, but no group so defined was at particularly high risk with respect to cancer in the mothers. Among all the mothers whose health status was known, there was a 3-fold excess risk ($P=0.01$).

Among the mothers of various sub-groups of children shown in the table, the highest relative risk of developing breast cancer was seen among the mothers of boys who were less than the median age at diagnosis, and who had pelvic tumours. In this group there were 0.3 cases of breast cancer

**Table III** Carcinoma breast in mothers of children with soft tissue sarcoma

| *Mother* Histology | Site | Age | *Child* Histology | Site | Age | Sex |
|---|---|---|---|---|---|---|
| Infiltrating duct | L | 27 | Dense poorly differentiated rhabdomyosarcoma | Middle ear | 1 | M |
| Infiltrating duct | L | 39 | Dense poorly differentiated rhabdomyosarcoma | Perianal | 3 | F |
| Infiltrating lobular | R | 42 | Loose botryoid rhabdomyo-sarcoma | Bladder | 1 | M |
| i) Tubular<br>ii) Tubulo-lobular | L<br>R | 44<br>45 } | Loose non-botryoid rhabdo-myosarcoma | Bladder | 1 | M |
| Infiltrating lobular | L | 46 | Fibrosarcoma | Pelvis | 3 | M |
| i) Mixed infiltrating duct/infiltrating lobular<br>ii) Infiltrating duct | L<br>R | 53<br>55 } | Malignant fibrous histiocytoma | Pelvis | <1 | M |

**Table IV** Other malignancy in mothers of children with soft tissue sarcoma

| *Mother* | | | *Child* | | | |
|---|---|---|---|---|---|---|
| *Histology* | *Site* | *Age* | *Histology* | *Site* | *Age* | *Sex* |
| Anaplastic malignant glioma | Frontal & temporal lobe | 33 | Dense moderately differentiated rhabdomyosarcoma | Perianal | <1 | M |
| Lymphoma, diffuse centroblastic/centrocytic | Cervical lymph nodes | 45 | Dense poorly differentiated rhabdomyosarcoma | Thigh | 12 | M |
| Endometrioid carcinoma | Bilateral ovary | 46 | Embryonal sarcoma | Bladder | 3 | F |
| Carcinoma of non-keratinizing type | Post-nasal space | 56 | Dense poorly differentiated rhabdomyosarcoma | Thoracic wall | 11 | M |
| Well differentiated adenocarcinoma | Sigmoid colon | 55 | Embryonal sarcoma loose and dense areas | Mediastinum | 4 | F |
| Well differentiated adenocarcinoma | Endometrium | 58 | Embryonal sarcoma | Pelvis | 5 | F |

**Table V** Excess risk of cancer among mothers of various sub-groups of children with soft tissue sarcoma

| | | *Cancers in mother* | | | | | | | |
|---|---|---|---|---|---|---|---|---|---|
| | | *Breast cancer* | | | | *Other cancer* | | | |
| *Sub-groups of children* | (*No.*) | *Expected number* | *Observed number* | *R.R.* | *P* | *Expected number* | *Observed number* | *R.R.* | *P* |
| All cases where mother's health status known | (143) | 2.0 | 6 | 3.0 | 0.01 | 4.5 | 6 | 1.3 | 0.2 |
| Less than median age at diagnosis | (71) | 0.8 | 6 | 7.6 | 0.0001 | 1.7 | 2 | 1.2 | 0.4 |
| Genito-urinary or other intra-pelvic tumour | (45) | 0.7 | 5 | 7.5 | 0.0004 | 1.5 | 3 | 2.1 | 0.1 |
| Male | (90) | 1.2 | 5 | 4.2 | 0.01 | 2.6 | 3 | 1.2 | 0.4 |
| Less than median age at diagnosis, with genito-urinary or other intra-pelvic tumour | (35) | 0.5 | 5 | 10.8 | 0.0001 | 1.0 | 2 | 2 | 0.2 |
| Male, less than median age at diagnosis, with genito-urinary or other intra-pelvic tumour | (25) | 0.3 | 4 | 13.5 | 0.0001 | 0.6 | 1 | 1.6 | 0.3 |

expected, and 4 were observed. The relative risk was 13.5 ($P=0.0001$). Among the corresponding sub-groups, for example girls and children with tumours at other sites, the expected numbers of breast cancer did not differ significantly from the numbers observed. The numbers of other cancers found among these mothers was only slightly in excess of the expected number, and was not statistically significant (relative risk 1.3; $P=0.2$). There was no association of these other cancers with the mothers of any particular sub-group of children. For the calculation of these relative risks, bilateral tumours were considered to be one case, although in some circumstances bilateral tumours may be registered separately in the Regional Cancer Register. The relative risks shown in Table V are therefore slightly *under*-estimated.

In addition to the malignant tumours shown in the tables, 5 other cases of neoplastic disease occurred amongst the mothers: 2 cases of uterine fibroids, fibroma of the lung, lipoma of chest wall and cervical carcinoma *in situ*. Since no population data are available for these tumours the significance of the 5 cases cannot be estimated.

### Discussion

In this population-based study, the mothers of children with soft tissue sarcoma experienced a significantly increased incidence of carcinoma of the breast, compared with the general female population living in the same geographical region. The overall risk to the mothers was 3-fold, but in the present study population the mothers of children in a particular sub-group defined by age of the child at diagnosis, the primary site of the soft tissue sarcoma, and the sex of the child, were at substantially greater risk. It may be, therefore, that among childhood soft tissue sarcomas there are two fractions: one where inherited factors are the most important element in aetiology, and a second fraction where the genetic role is less marked. Mothers of children in the first fraction would be at greatest risk.

Previous reports of the association of breast cancer with soft tissue sarcoma in families have been anecdotal, and the families identified because of the coincidence in time of 2 or more cases. The present study indicates that the genetic fraction among childhood soft tissue sarcoma may be greater than hitherto suspected. In the literature 5 families have been described where the mothers of children with soft tissue sarcoma had breast cancer, and where the age of the child at diagnosis, the site of the primary tumour and the sex of the child is given. Among these families there were 7 children with soft tissue sarcoma – 3 boys and 4 girls. All of the children were 3 years of age or under at diagnosis, 2 of them had pelvic tumours, 1 had a tumour of the eye, 1 had a tumour of the paranasal region and 3 had tumours of limbs (Li & Fraumeni, 1982; Pearson *et al.*, 1982; Duncan & Miller, 1983). Comparing these findings with the present series it may be that age at onset in the child is the most important indicator of a possible familial trait. Combining children reported in the literature with those in the present series 7 (54%) had pelvic tumours, which is a similar proportion to the group as a whole.

The distribution of histologies seen in the breast cancers among these mothers was unusual. In an unselected series of 1068 patients with breast cancer presenting to a local hospital 40 (3.7%) had bilateral disease, giving a total of 1108 cancers. The histology of these cancers was reviewed by one of us (MH) and only 9% were lobular compared with 81% infiltrating duct. It is not possible to compare the histology of the breast cancers found in the present series with that of similar families previously reported, because this information is not available. However, histology has recently been studied among the 31% of a series of 1024 women consecutively treated for breast cancer who reported at least one close female relative with breast cancer (Rosen *et al.*, 1982). In this study no single histological type was consistently associated with a high frequency of breast cancer in all classes of relatives, although the highest frequency of breast cancer among their sisters was found in patients with lobular carcinoma. Bilaterality and early age at onset have also been linked with familial breast cancer (Lynch, 1981). The occurrence of 2 cases of bilateral breast cancer out of a total of 6 among the present series would support a genetic aetiology. An association of bilaterality with a high proportion of lobular and tubular carcinoma of the breast has been noted by some workers (Robbins & Berg, 1964; Finney *et al.*, 1972). Of the 80 cancers occurring in the 40 patients with bilateral disease described above 19% were lobular. In the 2 mothers with bilateral disease in the present series, 2 of the 4 cancers showed some lobular features and 2 showed tubular features, 1 being tubulo-lobular. This is especially interesting in the context of the suggestion that tubular carcinoma is histogenically related to lobular carcinoma (Eusebi *et al.*, 1979).

It has been suggested that familial breast cancer has a better prognosis than sporadic (Albano *et al.*, 1982). Among the present 6 cases, 2 are still alive 20 years and 19 years respectively after diagnosis; a third patient is alive nearly 3 years after diagnosis; and a fourth patient died after $7\frac{1}{2}$ years. The remaining 2 cases died within 2 years of diagnosis. The crude survival rate for breast cancer in England and Wales, 1971–1973, for females aged between 35 and 54, was 59%–63% at 5 years (Toms, 1982). Accurate assessment of survival amongst hereditary cases of breast cancer compared with sporadic cases requires larger series and standardization for age, histology and stage at presentation.

All 6 women with breast cancer in the present series were pre-menopausal at diagnosis, as confirmed from their medical records, and were young compared with a median age at onset in the population of between 60 and 64 years (North Western Regional Health Authority, 1982). This apparent early onset may be a reflection of the age structure of this series of women. A follow-up of these mothers over a number of years may reveal the development of further cases of breast cancer as the women become older, and thus the average age at onset may increase. These findings indicate the need to take an adequate family history from new cases of breast cancer, particularly those with bilateral disease and/or premenopausal onset. Histological type may also be an indicator of inherited disease. Recognition that the occurrence of early onset cancer in close relatives could indicate heritability may lead to the identification of other women at risk with the possibility of early detection and treatment.

The 6 other cancers to occur amongst this series of mothers were mainly of unusual histological types. The number of cases did not exceed expectation, and therefore the array of histologies seen may be a reflection of the age distribution of this population of women.

The present study was specifically concerned with the occurrence of malignant disease among the mothers. However it is likely that other family members may also be at risk of developing malignant disease, and in this context in the present series six of the children had siblings with tumours, including one sib-pair where both children had soft tissue sarcomas. The other siblings had adrenal cortical tumour, Wilms' tumour, and two cases of astrocytoma respectively. The mother of the child whose sib had an adrenal cortical tumour developed breast cancer at age 27 (Table III). This family has previously been reported (Pearson *et al.*, 1982), and is a clear example of the syndrome described by Li & Fraumeni (1969).

The maternal grandmother of the child whose mother had a glioma (Table IV) is reported to have died of breast cancer at the age of 42 years. This is of particular interest since brain tumours are also a common feature of the "Li–Fraumeni" type families described in the literature. Furthermore, the index child fits into the sub-group whose mothers are at greatest risk for breast cancer as found in the present study. It may be that in this family the various cancers have a mainly genetic aetiology and this mother accounts for the slight excess of cancers other than breast found among the whole group of mothers.

Clearly there is a need to obtain pedigree histories from all of the families included in the study in order to determine more accurately the proportion of childhood soft tissue sarcomas which have a predominantly genetic aetiology.

Some cancer-prone conditions, for example xeroderma pigmentosum and ataxia telangectasia, characteristically show *in vitro* sensitivity to killing by UV light and gamma radiation respectively in cells from patients with these disorders. Conversely, cells from 2 patients who were members of the family described by Blattner *et al.* (1979) showed *in vitro* resistance to cell killing by gamma radiation (Bech-Hansen *et al.*, 1981). Further *in vitro* studies on material obtained from members of such families are obviously needed and may help to elucidate patterns of inheritance by identifying asymptomatic gene carriers in addition to contributing to knowledge of mechanisms of inheritance and malignant transformation at the cellular and molecular level.

In conclusion, we have found a significantly increased risk of developing breast cancer in the mothers of children with soft tissue sarcomas, and this excess risk is particularly marked in a certain sub-group. It may therefore be possible to recognize families at greatest risk at the time of the child's diagnosis even in the absence of a positive family history of malignant disease at that time. Further pedigree studies of the group as a whole, and *in vitro* studies among selected members of the families, may clarify this aspect. This work is important in that it offers the basis for more enlightened genetic counselling to be given to families of children with soft tissue sarcoma, selecting patients who might benefit from screening with the prospect of early diagnosis and more effective treatment in those members of the families at greatest risk. In addition, the work may contribute to the understanding of the genetic component in the aetiology of these diseases.

We are most grateful to the general practitioners who kindly supplied information to this study, and to staff of the National Health Service Central Registry, Southport, and of the Family Practitioner Committees for their co-operation. We also wish to thank the pathologists who willingly sent us material for review.

The Manchester Children's Tumour Registry is supported by Cancer Research Campaign.

### References

ALBANO, W.A., RECABAREN, J.A., LYNCH, H.T. & 5 others. (1982). Natural history of hereditary cancer of the breast and colon. *Cancer*, **50**, 360.

BECH-HANSEN, N.T., BLATTNER, W.A., SELL, B.M. & 4 others. (1981). Transmission of in vitro radioresistance in a cancer-prone family. *Lancet*, **i**, 1335.

BIRCH, J.M., MARSDEN, H.B. & SWINDELL, R. (1980). Incidence of malignant disease in childhood: A 24-year review of the Manchester Children's Tumour Registry data. *Br. J. Cancer*, **42**, 215.

BLATTNER, W.A., McGUIRE, D.B., MULVIHILL, J.J., LAMPKIN, B.C., HANANIAN, J. & FRAUMENI, J.F., Jr. (1979). Genealogy of cancer in a family. *JAMA*, **241**, 259.

BOTTOMLY, R.H., TRAINER, A.L. & CONDIT, P.T. (1971). Chromosome studies in a "cancer family". *Cancer*, **28**, 519.

DUNCAN, M.H. & MILLER, R.W. (1983). Another family with the Li–Fraumeni cancer syndrome. *JAMA*, **249**, 195.

ENZINGER, F.M. & WEISS, S.W. (1983). *Soft Tissue Tumors*. C.V. Mosby Co., St. Louis. Ch. 15.

EUSEBI, V., BETTS, C.M. & BUSSOLATI, G. (1979). Tubular carcinoma: a variant of secretory breast carcinoma. *Histopathology*, **3**, 407.

FINNEY, G.G., Jr., FINNEY, G.G., MONTAGUE, A.C.W., STONESIFER, G.L., Jr., & BROWN, C. C. (1972). Bilaterial breast cancer, clinical and pathological review. *Ann. Surg.*, **175**, 635.

LECK, I., BIRCH, J.M., MARSDEN, H.B. & STEWARD, J.K. (1976). Methods of classifying and ascertaining children's tumours. *Br. J. Cancer*, **34**, 69.

LI, F.P. & FRAUMENI, J.F., Jr. (1969). Soft-tissue sarcomas, breast cancer and other neoplasms. A familial syndrome? *Ann. Int. Med.*, **71**, 747.

LI, F.P. & FRAUMENI, J.F., Jr. (1975). Familial breast cancer, soft-tissue sarcomas and other neoplasms. *Ann. Int. Med.*, **83**, 833.

LI, F.P. & FRAUMENI, J.F., Jr. (1982). Prospective study of a family cancer syndrome. *JAMA*, **247**, 2692.

LYNCH, H.T., (Ed.) (1981). *Genetics and Breast Cancer*. Van Nostrand Reinhold Co. New York.

LYNCH, H.T., MULCAHY, G.M., HARRIS, R.E., GUIRGIS, H.A. & LYNCH, J.F. (1978). Genetic and pathologic findings in a kindred with hereditary sarcoma breast cancer, brain tumors, leukemia, lung, laryngeal, and adrenal cortical carcinoma. *Cancer*, **41**, 2055.

NORTH WESTERN REGIONAL HEALTH AUTHORITY. REGIONAL CANCER REGISTRY (1982). *Cancer in the North West Statistics for 1975–1978*.

PEARSON, A.D.J., CRAFT, A.W., RATCLIFFE, J.M., BIRCH, J.M., MORRIS JONES, P. & ROBERTS, D.F., (1982). Two families with the Li-Fraumeni cancer family syndrome. *J. Med. Genet.*, **19**, 362.

ROBBINS, G.F. & BERG, J.W. (1964). Bilateral primary breast cancers. A prospective clinicopathological study. *Cancer*, **17**, 1501.

ROSEN, P.P., LESSER, M.L., SENIE., R.T. & KINNE, D.W. (1982). Epidemiology of breast carcinoma III. Relationship of family history to tumor type. *Cancer*. **50**, 171.

ROTHMAN, K.J. & BOICE, J.D., Jr. (1982). *Epidemiologic Analysis with a Programmable Calculator*. Epidemiology Resources Inc., Boston.

TOMS, J.R. (1982). *Trends in Cancer Survival in Great Britain*. Cases registered between 1960 and 1974. Cancer Research Campaign, London.